# Positive Margins Following Excision of Primary Bone & Soft Tissue Tumours in a Tertiary Centre and the Impact on Patient Outcomes

**DOI:** 10.7759/cureus.21235

**Published:** 2022-01-14

**Authors:** George Matheron, Amir Ardakani, Ahmad Nasir, Panagiotis Gikas

**Affiliations:** 1 Sarcoma & Joint Reconstruction Unit, Royal National Orthopaedic Hospital, London, GBR

**Keywords:** soft tissue tumours, bone tumours, recurrence, morbidity, margins of excision, sarcoma

## Abstract

Background

Primary bone and soft tissue sarcoma treatment includes surgical resection, with or without peri-operative chemoradiotherapy. The aim of surgery is to achieve complete excision, to prevent localised recurrence and achieve cure. For various reasons, excision with adequate margins is not always possible. Our aim is to assess the occurrence of unexpected positive margins following primary excision within a tertiary centre and the impact on patient outcomes.

Methods

A retrospective analysis of 567 patients discussed at the Royal National Orthopaedic Hospital Multi-disciplinary team (MDT) meeting with positive margins between 1999-2020 was performed. Exclusion criteria included: excisions performed externally and lesions treated with curettage. Information gathering from electronic records highlighted 23 cases with unexpected positive margins following primary excision.

Results

All patients pre-operatively expected to achieve complete primary resection. The median age was 60 years (8-92), 10M:13F. Tumour location included lower limb (12), upper limb (six), pelvis (two) and trunk (three); eight bone tumours and 15 soft tissue. The overall recurrence rate was 30.4% (7/23). In those recommended for re-excision (n=16), the recurrence rate was 31.25% (5/16). Of the patients not initially recommended for re-excision (n=7), four proceeded to surveillance alone with 50% recurrence (2/4), both with metastatic disease not surviving to follow-up. A further three patients underwent post-operative radiotherapy alone with no recurrences at follow-up, one patient not surviving for further treatment due to stroke. The mean follow-up for patients was 3.1 years.

Conclusion

When positive margins do occur unexpectedly, the impact due to the need for further treatment and ultimately increased risk of recurrence can be significant. Results can be compared to those for unplanned excisions. Therefore, surgeons should be aware of the different circumstances in which positive margins occur to help guide treatment planning and managing patient expectations.

## Introduction

The standardised treatment of primary bone and soft tissue sarcoma includes surgical resection, either in isolation or accompanied by peri/post-operative radiotherapy with or without the addition of chemotherapy. The primary aim of surgical treatment is for complete excision to prevent localised recurrence, metastasis and ultimately achieve disease control [1]. Surgical margins can be histologically described as clear, marginal or positive, the latter being either planned or unplanned. There is no clear consensus on the exact surgical margin required to prevent local recurrence, but it has been well established that marginal or positive margins are associated with increased recurrence risk [2-4]. Owing to the rarity of bone and soft tissue sarcomas, some of these tumours are often unknowingly resected without prior investigation or multi-disciplinary team involvement. In such cases, in the absence of appropriate planning and intent to achieve tumour-free margins, there is often a significant residual tumour burden left behind, increasing the likelihood of local recurrence and the need for further treatment [5,6].

There are also occasions in the treatment of patients with primary bone and soft tissue sarcomas where complete excision with adequate margins is not always possible, even in those who have had appropriate pre-operative multi-disciplinary team planning. This is particularly relevant following the drive to achieve limb salvage surgery, where anatomical considerations and constraints prevent complete surgical excision [7]. Patient preference can also alter the recommended course of treatment, for example, when an amputation is declined. The alternative option can sometimes include offering surgery with an accepted marginal excision or planned positive margin. It is also important to take into consideration factors related to the biological nature of specific sarcoma subtypes that influence the requirement for adjuvant therapy, as well as the likelihood of recurrence requiring further treatment.

A sarcoma that was intended for complete resection but is later found to be histologically positive in its margins is sometimes referred to as an unplanned positive margin [8].

The objective of this study was to evaluate the occurrence of unplanned positive margin following excision of primary bone and soft tissue sarcoma within a tertiary referral centre in patients who underwent planned surgery through a multi-disciplinary approach. We will also analyse the impact on patient outcomes and survival, as well as explore the possible techniques that can be implemented to reduce the occurrence of unplanned positive margins.

## Materials and methods

Data was collected retrospectively using the Royal National Orthopaedic (RNOH Stanmore, UK) histological database combined with the sarcoma multi-disciplinary team (MDT) data set. Patients coded as having positive surgical margins following resection of bone & soft tissue sarcomas were identified. Previous studies [8] have classified positive margins into four groups based on different clinical circumstances. In this study, we focussed on those considered ‘group four’ patients; with unplanned positive margins following primary resection. A true positive margin was categorised as those with evidence of tumour cells present at histological examination. As unplanned positive margins, there were no patients with positive macroscopic margins intra-operatively.

Our exclusion criteria included; patients who had undergone unplanned excisions at external sites prior to referral to a tertiary centre, lesions treated with curettage and therefore by definition intralesional, those undergoing neoadjuvant chemotherapy, and those without a named Stanmore consultant (three patients). This yielded 23 patients from the original database of 567 patients. The following data was then subsequently extracted from electronic patient records: age, gender, location of the primary tumour, histological subtype (assessed by pathologists with expertise in sarcoma as part of the MDT approach), presence of metastasis at presentation, expected margin prior to excision, peri-operative treatment, outcome of MDT following resection (including margin status), and the need for further management and disease-free survival at the time of data collection.

## Results

From the time period January 1999 to June 2020, 567 patients were coded as having positive surgical margins following resection of bone and soft tissue sarcomas. Applying the exclusion criteria set out above, 23 patients met the inclusion criteria for further analysis (Table 1).

**Table 1:** Details of 23 patients with positive margins following primary bone or soft tissue sarcoma resection

Variable	Subgroup	Number
Demographics	Mean age (years)	60 (8-92)
	Sex (M:F)	10:13
Mean follow-up (years)		3.1
Type	Bone	8
	Soft tissue	15
Location	Upper limb	6
	Lower limb	12
	Pelvic girdle	2
	Trunk	3
Grade	1	2
	2	7
	3	14
Evidence of metastasis at presentation	Yes	1
	No	17
	Indeterminant (metastasis at follow-up)	5 (2)
Initial surgical management	Local excision	15
	Distal femoral replacement	2
	Proximal femoral replacement	3
	Sacrectomy	1
	Hemi-pelvectomy	1
	Sub-total scapulectomy	1
Recurrence rate	Overall	30.4% (7/23)
	Recommended for re-excision	31.25% (5/16)
	Following re-excision	26.66% (4/15)
	Following Post-excision radiotherapy alone	0% (0/3)
	Surveillance alone	50% (2/4)
Final status	Alive, disease-free	15
	Alive, disease present	3
	Died from disease	4
	Died from other causes	1

Of the 23 patients subsequently reviewed, the initial pre-operative recommendation from multi-disciplinary planning was that all 23 patients were expected to achieve complete local resection. The median age of the patients analysed was 60 years (8-92), 10 Male: 13 Female. Tumour location included lower limb (12), upper limb (six), sacrum/pelvis (two) and trunk (three). Eight patients presented with primary bone tumours, and soft tissue in the remaining 15, with 13 differing histological variants (Table 2). Of these, the initial operative intervention included local soft tissue excision (15), distal femoral replacement (two), proximal femoral replacement (three), sacrectomy (one), hemipelvectomy (one) and sub-total scapulectomy (one). All 23 unplanned positive margins were subsequently discussed in follow-up MDT meetings. The recommendation was made for 16 patients to undergo further surgical intervention, one patient was unable to proceed due to surgical site infection. Of the remaining patients, four proceeded to surveillance alone, and three were advised for post-operative radiotherapy (RT).

**Table 2:** Histological subtypes of tumours encountered

Subtype	Number of patients
Osteosarcoma	2
Chondrosarcoma	4
Synovial sarcoma	4
Spindle cell	1
Myxofibrosarcoma	5
Angiosarcoma	1
Clear cell	1
Leiomyosarcoma	3
Liposarcoma	2

The overall recurrence rate following an unplanned positive margin was 30.4% (7/23). Of these, one patient had undergone pre-op RT, two post-op RT and two had no adjunct treatment, whilst two patients were unable to proceed to further treatment for medical reasons.

In those recommended for re-excision (n=16), the recurrence rate was 31.25% (5/16). Fifteen of these patients proceeded to re-excision with a recurrence rate of 26.66% (4/15). Following re-excision, two (13.3%) of the 15 patients undergoing re-excision were found to have a positive repeat margin on histological examination, both suffering recurrence and subsequent death.

When assessing those tumours which recurred (n=7), one was of bony origin, the remainder soft tissue sarcomas: one grade 1, three grade 2 and three grade 3. Patient ages spread from eight years to 92 years, with no predilection for recurrence with increasing age. Following initial staging at presentation, one patient was found to have metastasis, one with indeterminate lung nodules (later found to be metastasis), and the remainder no metastatic disease. Pre-operative tumour dimensions were reviewed with little difference between those with or without recurrence. In recurring tumours, the mean maximum dimension was 8.47cm (range 1.2-17.2), whilst in those without further recurrence was 7.83cm (range 1.6-17.2). 

Of the patients not initially recommended for re-excision following MDT discussion (n=7), four proceeded to surveillance alone with 50% recurrence (2/4), both were found to have a metastatic disease not surviving to follow-up. In those patients proceeding to surveillance alone; one patient was elderly and co-morbid, therefore in discussion with the patient, it was deemed to be in their best interests to have no further invasive treatment, one patient refused additional treatment, and in the remaining two patients adjuvant therapy was deemed unlikely to reduce recurrence and instead to instigate further treatment if recurrence occurred. A further three patients underwent radiotherapy alone following primary excision with no recurrences at the time of data collection, one patient did not survive for further treatment due to a stroke. The mean follow-up for patients in the study was 3.7 years (range 0.27-7.4 years).

## Discussion

An experienced multi-disciplinary specialised sarcoma group with access to appropriate pre-assessment as well as the full range of surgical and adjuvant treatment is key to the successful management of sarcomatous tumours. Within this setting, aiming to achieve negative margins is the core of any oncological surgery [9]. However, the relative rarity and heterogeneity of bone and soft tissue sarcomas, occurring virtually anywhere in the body, can make their surgical management challenging. In spite of expert sarcoma centre management, The Scandinavian Sarcoma Group report crude local recurrence rates of 17% [10].

Due to the varied location and common association to important neurovascular structures, it is often challenging for surgeons as part of the MDT to decide on the likelihood of successful primary excision within the context of limb salvage. The desire to preserve function, however, may have an impact on the decisions made on surgical margins pre-operatively.

Following on from Enneking’s original work, various authors have described methods of the classification of tumour margins, as well as suggesting what margins can be classified as sufficient for disease control [11-14]. A definitive answer remains elusive, and therefore the question remains, how close is close enough?

It is well established that the occurrence of positive margin surgery in the primary excision of soft tissue sarcoma (STS) has a significant impact on local and distant recurrence, particularly when compared to patients with negative margins, in which a high level of disease control can be demonstrated [15,16].

When microscopic positive margins are discovered following surgical excision, it has been suggested that the circumstances in which these occur may provide predictive value as to ongoing disease control, morbidity and survival. This has important consequences for the patient and treating team [4,8,16].

A study by Gerrand et al., considering the effect of positive margin surgery following primary excision in limb STS, showed a five-year local recurrence rate of 36.6%, with no significant difference between unplanned resection and unplanned positive margin in crude local recurrence rate, as well as local recurrence-free survival and disease-specific survival [8].

Further work by O’Donnell et al. highlighted a local recurrence rate of 34.8% in patients with unexpected positive margins following primary excision. There was a significant associated reduction in five-year local recurrence-free survival and cause-specific survival compared to a negative margin control group [16].

Following on from this, we used the previously described classification of positive margins to further explore the effect of unplanned positive margin after primary resection on patients at our centre [8,16]. A crude recurrence rate of 30.4% is comparative. Further comparing to recurrence rates in unplanned excisions shows no juxtaposition, affirming the significant impact that unplanned margins even in planned surgery has on local recurrence (LR) [8,17].

When positive margins do occur unexpectedly, pre-operative treatment plans are re-assessed through the multi-disciplinary team. This often necessitates further surgery or adjuvant therapy with the aim of achieving subsequent negative margins, and therefore minimising recurrence risks. It is also important to consider that further excision may need to be more radical to ensure complete resection, incurring patient morbidity [4,9,17]. This was certainly highlighted within our patient group, with 15 of 23 proceeding to further surgery and 11 to further adjuvant therapy. Certainly, in some patients, there may be one chance to achieve a negative margin due to medical or surgical considerations preventing further treatment. This meant that three patients in our study were not able to undergo further treatment, all of whom subsequently died.

The occurrence of unplanned positive margin surgery is multifactorial. Firstly, surgical factors, including surgical error, may represent that true margins can be difficult to accurately assess intra-operatively, rather than simply surgical skill. Secondly, whilst cross-sectional imaging plays a key role in surgical planning, the true margins of the tumour may extend beyond those identified [18]. This may particularly be the case if there is a delay between imaging and intervention. Thirdly, papers by O’Donnel et al., based on earlier work by Kawaguchi et al., suggest five-year cause-specific survival rates are only slightly improved by resecting tumours encroaching on critical structures en-bloc to achieve negative margins, compared to critical structure-preserving planned positive margins. In contrast, both groups were significantly worse off than the negative margin group. This work suggests that although negative margin surgery remains a keystone, tumours surrounding and invading critical structures could represent an innate biological aggressiveness, leading to increased local recurrence in spite of negative margin status. It is, however, important to delineate from those not surrounding critical structures but positive in the soft tissues surrounding the tumour, in which outcomes are worse for patients.

Limitations

There are several limitations to the current study. Firstly, data was collected retrospectively, covering a greater than 20-year period. As documentation and recording methods change over time, ensuring that the appropriate data has been captured, as well as assessing any surgical factors leading to positive margin surgery can be challenging. Secondly, and associated with the time periods involved, the relatively small number in the overall cohort due to the relative infrequency of sarcomas, with a smaller cohort remaining where true unplanned positive margins occur [16]. However, the numbers presented here are in line with studies presented previously on this topic [8,16]. Thirdly, there remains no widely accepted definition of positive margin for sarcoma excision. In our study, we define this to be a microscopic positive margin in resected specimen, as in the American Joint Committee on Cancer/International Union Against Cancer. Although definitions vary - direct comparisons can be challenging. 

What can be done to reduce the incidence of unplanned positive margins?

Reducing the risk of unplanned positive margin surgery is unlikely to be achieved by any single approach, reflecting the complexity of sarcoma management. However, we believe that there are several approaches that can be considered to minimise the risk.

The first key step following referral remains careful multi-disciplinary planning prior to primary excision. Within the MDT, documentation should be clear to highlight when marginal or critical structure margins are expected. The likelihood of complete resection should be carefully considered, remaining conservative regarding the possibility of success. In the context of increased limb-sparing surgery, more radical surgery should always be considered and discussed with patients with the knowledge of the impact of positive margins on morbidity and mortality.

Cross-sectional imaging alongside expert advice from radiologists is used to assess the extent of the tumour, guiding surgical margins. Variability of tumour aggressiveness should be considered; minimising the time between any imaging used in the pre-operative planning of margins and surgery to reduce the risk of incorrect assessment.

Operatively, several methods could be considered to limit risk. These include dual consultant operating where possible for more difficult cases, the introduction of stop checks of surgical margins, and dual instrument measurements.

Future technology could also be used to enhance the accuracy of resection, for example, the use of 3D printed technology to produce custom jigs as a resection guide.

## Conclusions

It is clear that positive margins can be a devastating complication for patients and their treating team. However, there is an increasing suggestion that not all positive margins are created equal. We suggest that unplanned positive margin surgery often leads to the need for morbid treatment, as well as carrying a significantly increased risk of recurrence. Therefore, surgeons should be aware of the different circumstances in which positive margins occur to help provide a guide to treatment requirements, as well as managing patient expectations.
